# NIHR statistics group imaging studies section: a network for statisticians and researchers using imaging in healthcare research

**DOI:** 10.1186/1745-6215-16-S2-P215

**Published:** 2015-11-16

**Authors:** Julia Forman, Susan J Dutton, Thomas R Fanshawe, Sue Mallett

**Affiliations:** 1 Cambridge Clinical Trials Unit, Cambridge University Hospitals, Cambridge, UK; 2 Centre for Statistics in Medicine & Oxford Clinical Trials Research Unit, Nuffield Department of Orthopaedics, Rheumatology and Musculoskeletal Sciences, University of Oxford, Oxford, UK; 3 Nuffield Department of Primary Care, University of Oxford, Oxford, UK; 4 Public Health, Epidemiology and Biostatistics, School of Health & Population Sciences, University of Birmingham, Birmingham, UK

## 

Incorporating imaging modalities into clinical trials and healthcare research presents particular challenges: What should be measured, and how? What summary statistic should be used? How can we best handle large amounts of multiple testing? How can reliability and misdiagnosis be assessed, and what are their implications?

The NIHR Statistics group (http://www.statistics-group.nihr.ac.uk/) aims to promote statistical methodology, provide educational opportunities, share best practice and develop a community of statisticians funded by NIHR research units or grants.

The NIHR Statistics group imaging Studies Section of the group provides a forum to address statistical issues in the design and analysis of imaging studies. The group aims to facilitate networking among statisticians, data analysts and other methodologists working in this area.

The Imaging Studies Section organises meetings every six months, with presentations on statistical challenges in imaging studies, small group discussions to share design ideas and expertise, and networking. A working group has been formed to organise meetings and facilitate related activities.

Our first meeting took place on the 22nd October 2014 in Oxford, attended by 25 members. This was followed by a second meeting on 27th April 2015 in Warwick, which focussed on inter-rater agreement and reproducibility of endpoint assessment within the context of clinical trials in Inflammatory Bowel Disease.

This poster will outline the key statistical issues that need to be addressed when designing and analysing studies that use medical imaging, and will also summarise the current and future plans of the Imaging Studies Section.

